# Editorial: The Regulation of Autophagy Activity in Pathogenic Microorganism Infection

**DOI:** 10.3389/fcell.2022.889283

**Published:** 2022-04-04

**Authors:** Shoulong Deng

**Affiliations:** National Health Commission of China (NHC) Key Laboratory of Human Disease Comparative Medicine, Institute of Laboratory Animal Sciences, Chinese Academy of Medical Sciences and Comparative Medicine Center, Peking Union Medical College, Beijing, China

**Keywords:** autophagy, pathogenic microorganism, immunity, viral infection, against bacterial

Autophagy, as an essential biological pathway with effects on immunity, is a powerful tool that host cells use to defend against bacterial and viral infection. Autophagy controls inflammation by interacting with innate immune signals, removing endogenous inflammasome agonists and affecting immune mediators to maintain cellular homeostasis. However, in an ongoing evolutionary arms race, several bacterial and viruses have acquired the potent ability to hijack and subvert autophagy for their benefit. In addition, the basic level of autophagy has a protective effect on cells, but excessive autophagy is associated with autophagic cell death and even accelerate the progression of the disease. Therefore, studying the regulation of autophagy activity during pathogenic microorganism infection can help us understand the mechanism of host disease-resistant microbial infection mechanisms. As the study develops in-depth, researchers have further found the close association between autophagy and immune systems, and autophagy is related to multiple innate immune responses in mammals. Normally, autophagy is also regarded as an original form of innate immune to defend against the invading microorganisms by activating pattern-recognition receptors that regulate various immune effects to clear cellular microbes with different autophagy adaptor proteins. Autophagy affects a variety of immune mediators to further regulate immune signaling pathways, which are associated with cellular environmental homeostasis by eliminating or presenting different intracellular material. This Research Topic aimed at the regulation of autophagy activity during different pathogenic microorganism infections, serving as the fundamental research in bacterial and viral diseases studies.

Liang et al. review the study’s progression on the function of autophagy in viral infection. On the one hand, host autophagy can orchestrate immunity to defend against viral infection. Its antiviral function mainly includes the following aspects. Firstly, host autophagy helps to degrade the targeting viral particles in autolysosomes. Secondly, host autophagy could activate innate antiviral immunity by promoting interferon production. Additionally, host autophagy participates in antigen presentation which further coordinates adaptive immunity. On the other hand, some viruses have evolved strategies to evade autophagy degradation or use autophagy to facilitate their replication. Liang et al. eloquently review how some viruses hijack and subvert autophagy to help their infection and pathogenesis. It is worth noting that Severe Acute Respiratory Syndrome Coronavirus-2 (SARS-CoV-2) evades autophagy by blocking the fusion of autophagosomes with lysosomes and inhibiting lysosome acidification. Moreover, SAR-CoV-2 spike hijack autophagy to induce abnormal inflammation and apoptosis.

Also, bacterial infections can cause a class of diseases that seriously endanger public health security. An interesting review by Wang et al. from the perspective of the relationship between oxidative stress and autophagy, fully describe their characteristics and connection, elucidate their effects on the male reproductive system and puts forward the corresponding countermeasures. To summarize, the mutual regulation and restriction of the oxidative stress-autophagy axis are critical to maintaining normal male fertility.

Mitochondria perform the functions of biological oxidation and energy exchange, and they also serve as signaling platforms in regulating apoptosis and host immunity upon infections. Mitochondrial homeostasis is crucial to maintaining host health. In most cases, mitochondrial metabolites or respiratory burst helps to eliminate the invading pathogenic organisms. However, abnormal mitochondrial metabolites help pathogenic organisms survive and trigger cell death. If the imbalance of mitochondrial homeostasis triggers autophagy or contributes to maintaining mitochondrial homeostasis remains controversial. Wang et al. review the relationship between autophagy and mitochondrial homeostasis during infection. Several signaling pathways, including AMPK signaling, cGAS-STING signaling, and TLR signaling, are involved in the links between mitochondrial homeostasis and autophagy. And these signaling affect autophagy initiation and mitochondrial dynamics. Mitophagy is considered as a selective autophagy process of mitochondrial degradation. Host innate antiviral immunity relates closely with mitophagy. Wang et al. fully describe how viruses manipulate mitophagy to escape from the surveillance of the host immune system. Mitophagy helps to dampen virus-induced type I interferon production and inflammation by inhibiting MAVS and cGAS-STING and affects the host adaptive immune system by regulating CD8-positive memory T cells activity. This review also enlightens some novel therapeutic strategies during viral infection.

An increasing number of target molecules have been identified as our understanding of autophagy, and pathogenic microorganism infection deepens. The initial studies of cGAS-STING mainly focus on their function in type I interferon induction, closely related to innate antiviral immunity. However, Gui et al. (2019) suggested the induction of autophagy may be a basic function of the cGAS–STING signaling pathways, but not type I interferon induction. Zhang et al. review the cGAS-STING signaling pathway in autophagy and type I interferon production. On the one hand, the activation of cGAS-STING signaling contributes to type I interferon induction and autophagy. On the other hand, autophagy also affects cGAS-STING activity, further regulating innate antiviral immunity *via* type I interferon. Huang et al. found *HMGB1* and *ATF3* play a critical role in autophagy and immune response to virus by a meta-analysis based on genome-wide association summary statistics during hepatitis virus infection. *HMGB1* participates in sensing hepatitis B virus (HBV) or hepatitis C virus (HCV) and further affects autophagy and inflammation. *ATF3* regulates the expression of autophagy gene Atg16l2 and cellular metabolism. This finding offers potential therapeutic targets for HBV/HCV infection.

In summary, this Research Topic has widened and deepened the understanding of autophagy’s role and regulation in pathogenic microorganism infection. Here, we thank all the authors who contributed to this work a lot. More importantly, their papers enlighten potential novel therapeutic strategies to improve public health security.
